# A case report of cerebral vasculitis induced by ipilimumab with nivolumab in a patient with metastatic renal cell carcinoma

**DOI:** 10.3389/fimmu.2026.1779015

**Published:** 2026-03-31

**Authors:** Patrik Palacka, Tomáš Málek, Marek Makovník, Renáta Felediová, Boris Rychlý, Georgína Kolníková

**Affiliations:** 1 2nd Department of Oncology, Faculty of Medicine, Comenius University and National Cancer Institute, Bratislava, Slovakia; 2 Cancer Research Institute, Biomedical Research Center, Slovak Academy of Sciences, Bratislava, Slovakia; 3 Department of Urology, University Hospital, Bratislava, Slovakia; 4 Department of Radiology, National Cancer Institute, Bratislava, Slovakia; 5 Department of Neurology, National Cancer Institute, Bratislava, Slovakia; 6 Department of Pathology, Unilabs, Bratislava, Slovakia; 7 Department of Pathology, National Cancer Institute, Bratislava, Slovakia

**Keywords:** cerebral vasculitis, metastatic renal cell carcinoma, non-clear-cell carcinoma, ipilimumab, nivolumab

## Abstract

The prognosis of patients with metastatic renal cell carcinoma (RCC) has improved with the introduction of novel combination therapies, including monoclonal antibodies such as ipilimumab (against CTLA-4) with nivolumab (against PD-1). However, these checkpoint inhibitors are associated with a broad spectrum of immune-related adverse events (irAEs). Herein, we report a case of suspected immune-related cerebral vasculitis occurring three weeks after initiation of ipilimumab plus nivolumab in a patient with metastatic not-otherwise-specified (NOS) renal cell carcinoma (RCC). Brain MRI demonstrated multifocal supratentorial cortico-subcortical lesions with diffusion restriction and focal cortical enhancement, compatible with acute to subacute ischemia. In the context of the clinical presentation, exclusion of common stroke mechanisms, and a favorable response to corticosteroids, both clinically and para-clinically with a marked decline in CRP, the findings were considered most consistent with possible immune-related cerebral vasculitis. Three months after only one application of combined immunotherapy, a restaging CT scan showed an objective response in terms of partial remission. However, the metastatic RCC progressed after another 3 months, and the patient received two lines of tyrosine kinase inhibitors (sunitinib and cabozantinib). The patient died due to cancer progression, with an overall survival of 10.5 months. This case report provides experience with the management of possible immune-related cerebral vasculitis in patients with a rare malignancy. Severe cerebral vasculitis requires hospitalization with prompt diagnostic workup and immunosuppressive treatment. Despite the preterm permanent discontinuation of immunotherapy, the patients can benefit from the systemic treatment.

## 1 Introduction

The current standard of care for patients with advanced clear cell renal cell carcinoma (ccRCC) is axitinib + pembrolizumab, cabozantinib + nivolumab, lenvatinib + pembrolizumab, or ipilimumab + nivolumab (1) regardless of their stratification according to the International Metastatic RCC Database Consortium (IMDC) prognostic model, which divides patients by the presence of risk factors (time < 1 year from diagnosis to initiation of systemic therapy, Karnofsky performance status < 80%, hemoglobin < lower limit of normal, corrected calcium > upper limit of normal, neutrophils > upper limit of normal, platelets > upper limit of normal) into three (favorable, intermediate, and poor) prognostic groups (2). Patients with a favorable prognosis have the option of receiving tyrosine kinase inhibitors (TKIs) sunitinib, pazopanib or tivozanib, and patients with an intermediate and poor prognosis have the option of receiving axitinib + toripalimab (1).

ChechMate214 is a phase 3 study demonstrating improved outcomes of patients with ccRCC receiving nivolumab + ipilimumab in the intention to treat (ITT) and the intermediate and poor prognosis (IPP) groups (3). At a median follow-up of 99.1 months, hazard ratios (HRs) for overall survival (OS) in the ITT and IPP groups were 0.72 and 0.69, respectively. The PFS probabilities for the ITT and IPP groups for 90 months were 22.8% and 25.4%, respectively. The objective response rate (ORR) was 39.5%, including 12.0% complete responses (CRs) for the ITT group, and 42.4%, including 11.8% CRs, for the IPP group. Immunotherapy-related adverse events (irAEs) and serious AEs (SAEs) occurred in 99.5% and 68.7% of patients, respectively. AEs led to therapy discontinuation in 23.6% of cases (4).

The not-otherwise-specified (NOS) subtype represents about 3–4% of all RCCs. This subtype is found where multiple variants or subtypes are seen in a single tumor. There could be a mixture of two to three different subtypes found. Unclassified RCC is sometimes associated with more aggressive disease and tends to be harder to treat (5). The efficacy and safety of nivolumab + ipilimumab in patients with advanced non-ccRCC, including the NOS subtype, was demonstrated in a randomized phase 2 study (6) and a phase 3b/4 study (7).

irAEs are organ-specific, with skin-related irAEs being the most common, followed by gastrointestinal toxicity, endocrine irAEs, and musculoskeletal and ocular toxicities. Pneumonia, myocarditis, neurotoxicity, myositis, nephritis, and hematological AEs are less frequent (8). Vasculitis is an uncommon presentation; the most frequently reported types are large vessel vasculitis and vasculitis of the nervous system. Developing vasculitis during therapy requires discontinuation, whether temporary or permanent (9).

The objective of this paper is to present a rare case of possible secondary immune-related cerebral vasculitis, which occurred three weeks following the first administration of ipilimumab + nivolumab in a patient with metastatic NOS RCC, because its diagnosis and treatment are challenging.

## 2 Case report

A 56-year-old patient, KB, without significant history or comorbidity, was admitted to the Department of Urology and underwent laparoscopic removal of the right kidney and adrenal gland due to a renal cell tumor. A pathologist (B.R.) evaluated the tumor as high-grade NOS RCC, classified as grade 4 by the International Society of Urological Pathology (ISUP). The resection margins were microscopically infiltrated with tumor cells (R1 resection). According to TNM, this patient was classified as pT4cN1M1.

Tissue specimens were fixed in 10% formaldehyde and processed into paraffin-embedded tissue using standard techniques. For immunohistochemical work, four-micrometer sections were cut from formalin-fixed paraffin-embedded (FFPE) blocks and stained using the Benchmark Ultra system (Ventana, Roche, Basel, Switzerland). Immunohistochemical stains showed positive staining for CK7, CD117, AE1/3, INI1, FH (fumarate hydratase), and CK8/18, as well as focal positivity for EMA and AMACR and negativity for PAX8. The commonly used immunohistochemical assay developed to determine the PD-L1 expression level was also performed (clone 22C3, Hérmes, Paris, France, DAKO, Glostrup, Denmark, OMNIS, Saxmundham, UK). The expression level was assessed using the combined positive score (CPS), which is calculated as the ratio of PD-L1 positive cells (including tumor cells, lymphocytes, and macrophages) to the total number of tumor cells x100. PD-L1 positivity was defined as a CPS of 1 or higher. The CPS in this case was 15%; the slides were independently reviewed by two pathologists (BR and GK).

**Figure 1:**
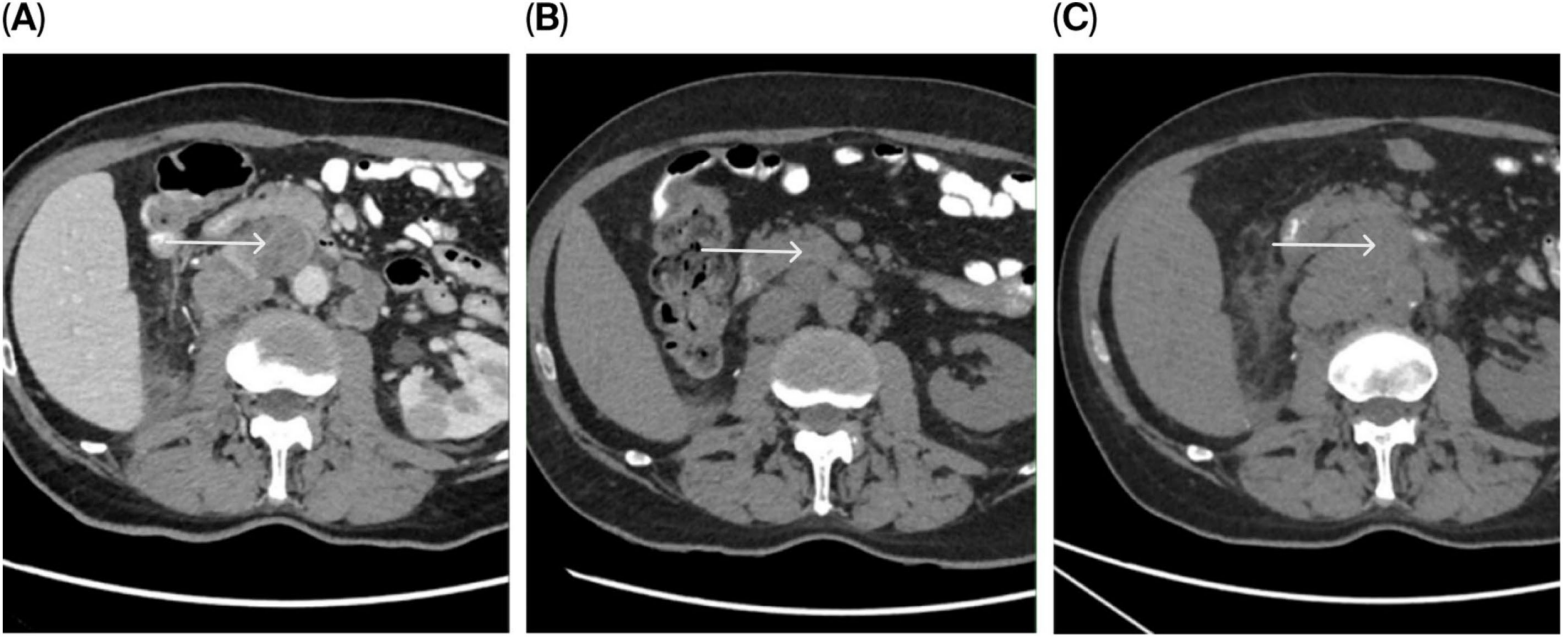
Immune response assessment by iRECIST on CT. **(A)** Baseline imaging was performed on 13 January 2025. **(B)** Follow-up was on 15 April 2025, showing a decrease in target retroperitoneal lesions consistent with iPR (the white arrow). **(C)** On 15 July 2025, an increase in tumor burden meeting RECIST 1.1 progression criteria was observed; according to iRECIST, this was categorized as *iUPD*.

The computed tomography (CT) scans, which were performed before systemic therapy initiation on 13 January 2025, showed small, non-specific nodules in the lungs, slightly enlarged mediastinal lymph nodes, multiple metastases in the liver, a single metastasis in the left adrenal gland, a duplex tumor in the dorsal labium of the left kidney, retroperitoneal lymphadenopathy (**Figure 1A**), a peritoneal metastasis and osteolytic metastases in L4, the right iliac bone, right scapula, right pubic bone, and right femur.

According to IMDC, this patient was stratified as poor risk. On 21 January 2025, he received a total dose of 100 mg of ipilimumab (1 mg per kg) + 240 mg of nivolumab intravenously, with 120 mg of denosumab subcutaneously. No acute complications were recognized.

On 8 February 2025, the patient was urgently hospitalized due to new-onset neurological symptoms, mainly consisting of facial drooping, disorientation, and unsteady gait. According to his family members, psychological changes such as depressive mood, loss of appetite, and gait instability have also appeared. Neurological examinations showed slow psychomotor tempo, lethargy, left-sided central cranial nerve VII (facial nerve) paresis, and postural instability. The duration of the patient*’*s symptoms and signs was no more than 48 hours before admission to the hospital.

**Figure 2:**
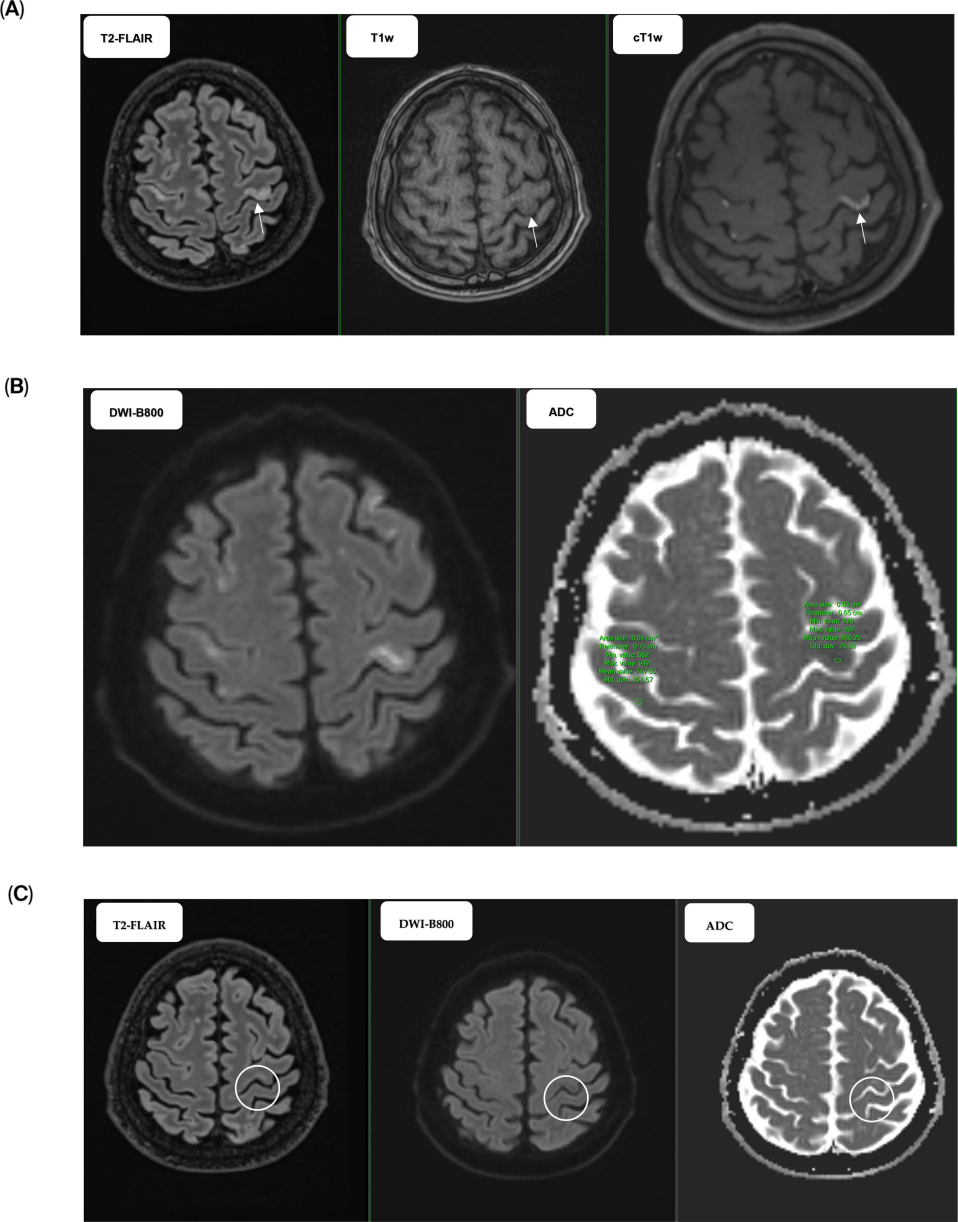
Secondary vasculitis following the administration of checkpoint inhibitors. **(A)** Brain MRI on February 14, 2025. Multiple T2-FLAIR hyperintense lesions in the supratentorial white matter and bilateral cortico-subcortical regions, with the most pronounced lesions in the frontoparietal region and relative sparing of the temporal lobes (arrow). No significant signal changes (arrow) on non-enhanced T1w images. Post-contrast T1w-black blood demonstrates cortical enhancement (arrow), indicating blood–brain barrier disruption. **(B)** Diffusion restriction is supported by objective high signal on DWI (b800) and ADC measurements demonstrating decreased ADC values within the lesions relative to normal-appearing contralateral parenchyma. The corresponding reduction is visible in the provided ADC map and ROI analysis. **(C)** Brain MRI on June 21, 2025. Follow-up MRI demonstrates regression of previously described T2-flair lesions (white circle) with restricted diffusion on DWI/ADC (white circle) with no new abnormalities. Contrast agent was not administered due to impaired renal function; therefore, assessment of enhancement was not possible.

Due to renal impairment, a CT angiography was not performed, but carotid and vertebral artery ultrasound did not show any extracranial stenosis, atherosclerosis or embolic sources indicative of stroke. A non-contrast CT showed no focal lesions, ischemia, or hemorrhage in the brain. Brain magnetic resonance imaging (MRI) revealed multiple T2 hyperintense lesions, which were localized diffuse bilateral supratentorial and cortico-subcortical with relative sparing of the temporal lobes in the white matter. The lesions showed diffusion restriction. Moreover, the cortical lesions evidenced postcontrast saturation. The rest of the examined anatomical structures showed no significant abnormalities. Magnetic resonance angiography time-of-flight (MRA TOF) results were negative. No significant vessel-wall signal abnormalities or pathological enhancement were demonstrated (**Figures 2A, B**). Paroxysmal atrial fibrillation was excluded using a Holter monitor. Transthoracic echocardiogram (TTE) was performed to exclude the cardioembolic sources and nonbacterial thrombotic endocarditis.

Laboratory tests included antinuclear antibodies (antiANA), antibodies against native double-stranded DNA (antidsDNA), antibodies against deoxyribonucleoprotein (antiDNP), antibodies against anticyclic citrulline peptide (antiCCP), perinuclear/cytoplasmic antibodies against neutrophil leukocytes (p/c ANCA), antibodies against myeloperoxidase (antiMPO), antibodies against proteinase 3 (antiPR3), antibodies against glomerular basement membrane (anti-BMG), antibodies against renal tubular basement membrane (anti-BMT), antibodies against PLA2-R-anti-phospholipase A2 receptor IgG (antiPLA2R), and screening for antibodies for antiphospholipid syndrome (APS).

Positive findings were found for anti-phosphatidyl and anti-phosphatidyl inositol antibodies, as well as anti-titin and anti-Ro-52, while polymyositis/scleroderma (PM-Scl75) antibodies were borderline. Cerebrospinal fluid (CSF) analysis was not performed by a neurologist due to patient refusal. The condition was assessed as a possible secondary cerebral vasculitis of grade 3 induced by immunotherapy based on the clinical course, laboratory results, and MRI findings.

Eosinophilia (22.7%, the absolute eosinophil count, AEC of 2.69), present before prednisone initiation at a dose of 1 mg per kg disappeared, and C reactive protein (CRP) levels declined from 124.7 to 46.5 mg/L within a week of initiating corticosteroid therapy. On April 15, 2025, the eosinophils, AEC and CRP were 0.9%, 0.06 and 27.80 mg/L, resp. Depending on the improvement of clinical conditions, prednisone was administered with gradual tapering and was discontinued on May 6, 2025, when the eosinophils, AEC and CRP were 3.8%, 0.3 and 25.40 mg/L, resp. During hospitalization stay, a low-molecular weight heparin (LMWH) nadroparin was given in prophylactic dose for 14 days only, blood pressure was controlled under 120/80 mm Hg adding perindopril in daily dose of 4 mg. A CT scan was performed on 15 April 2025 and showed a partial response (PR) in terms of regression of the retroperitoneal LAP by 15–20 mm (**Figure 1B**). There was a further reduction in the size of the mediastinal lymph nodes, the tumor in the left kidney, and the lesion in the left adrenal gland by 4–5 mm. The nodularity on the peritoneum disappeared, and the foci in the liver and bone metastases remained the same size. Brain MRI on 21 June 2025. Follow-up MRI demonstrates regression of previously described T2/flair lesions with restricted diffusion on DWI/ADC, with no new abnormalities. Contrast agent was not administered due to impaired renal function; therefore, assessment of enhancement was not possible (**Figure 2C**). Regarding neurological examination, there was an overall improvement of clinical condition, but persistent central paresis of the left cranial nerve VII, quadri-hyperreflexia and mild -spasticity with positive Babinski sign within the framework of pyramidal syndrome. Immunotherapy restart was discussed with the patient who preferred follow-up.

However, a CT scan performed in July revealed disease progression (**Figure 1C**). Due to national restrictions, we started second-line TKI sunitinib for 8 weeks without any significant AEs; however, further cancer progression was noted on CT scans. Additionally, obstructive jaundice was treated by inserting metallic stents into the right and left bile ducts. Between 24 September 2025 and 31 October 2025, he received cabozantinib, which was stopped due to disease progression verified by a CT scan. No serious AEs from cabozantinib were recognized. The patient passed away on 7 November 2025, with PFS on the three lines of therapy being 7 months, 2 months, and 1.5 months, respectively.

## 3 Discussion

This is a case report of a patient with metastatic non-clear cell RCC treated with ipilimumab and nivolumab who developed possible cerebral vasculitis as a serious AE of immunotherapy. This diagnosis was made *per exclusionem.* However, we must admit some limitations of this case including the absence of CSF analysis and additional angiographic or histopathological confirmation. The presence of phosphatidyl/inositol antibodies within APS-like state could explain multifocal embolic like infarctions, but symptoms and signs, which moderated shortly after corticosteroids tend to be in favor of immune-related vasculitis. Despite discontinuing systemic treatment, the cancer responded to the immune checkpoint inhibitors in terms of a partial remission. This is consistent with the positive correlation of responses to ipilimumab + nivolumab and the incidence of irAEs (10).

Vasculitis is a group of rare, clinically, and pathologically heterogeneous diseases mediated by the immune system. Their common feature is inflammation of the vessels, but they differ in the vessels affected, target organs, histology, clinical features, and treatment success. Ecclestone and colleagues published a proposed vasculitis classification system in 2023 (11), but not all immune-related inflammatory vascular diseases can be subsumed under one of the units within this classification. Vasculitis, as an adverse effect of immunotherapy, based on the inhibition of immune checkpoints, is rare, with an incidence of less than 1%. Its risk factors are unknown, and it has different clinical manifestations, severity, and treatment outcomes (12). The diagnostic method of choice is CT or MR angiography, with a characteristic image of diffuse circumferential thickening of the vessel wall with its enhancement or thrombus (13).

On MRI, cerebral vasculitis typically presents multiple patchy T2/FLAIR hyperintense lesions in the white matter and cortico-subcortical regions, often asymmetric and involving multiple vascular territories (14). Diffusion-weighted imaging frequently shows restricted diffusion corresponding to small-vessel ischemia not confined to a single arterial distribution. Post-contrast T1 sequences may demonstrate patchy or gyri form enhancement, reflecting disruption of the blood–brain barrier, while MR angiography or digital subtraction angiography can reveal segmental stenoses or a characteristic *“*beading*”* pattern in the cerebral vessels (15). In some cases, susceptibility-weighted imaging may additionally show cortical or subcortical microhemorrhages (16). Altogether, these findings support the diagnosis of vasculitis, particularly when the distribution and character of lesions are not typical for alternative processes such as demyelination or metastatic disease.

Due to the low incidence of vasculitis as an adverse effect of immunotherapy in patients with metastatic RCC, retrospective studies with larger numbers of patients are not available. Therefore, we could only analyze the diagnostics and treatment of vasculitis in the published case reports. The authors identified four such cases (17–20), which differ in terms of systemic therapy used (monotherapy *vs*. combined therapy), time of onset, clinical picture, localization of affected vessels, diagnostics (if biopsy was feasible), therapeutics, and checkpoint inhibitor(s) as a restart option.

In a patient with metastatic neuroendocrine RCC, treatment with nivolumab led to the development of eosinophilic granulomatosis with polyangiitis and systemic eosinophilic inflammation, causing repeated bronchial asthma attacks. A tissue sample obtained via bronchoscopy showed eosinophils infiltrating the small vessels in the lungs and severe alveolar hemorrhage, indicative of eosinophilic vasculitis and pneumonia. The corresponding findings were present on the CT scan, and immunosuppressive therapy with prednisolone was initiated at a daily dose of 20 mg. Once the dose was reduced to 10 mg, mepolizumab was added to the corticosteroid and nivolumab was restarted (17). Mepolizumab binds to the interleukin 5 (IL-5) receptor α subunit on the surface of eosinophils and prevents IL-5 from binding to its receptor. In the European Union, mepolizumab is approved as an add-on treatment for severe refractory eosinophilic asthma in adults (22).

In a woman with advanced RCC, myalgia and purpura on the limbs appeared following 18 months of nivolumab. Biopsy revealed leukocytoclastic vasculitis (LCV) with deposits of IgA and complement 3 (C3) in the vessel walls of the upper dermis. Oral prednisolone (20 mg daily) led to a complete disappearance of both clinical manifestations. After 3 months, nivolumab was reinitiated, but no AE relapse was recorded (18). In another woman with metastatic ccRCC, the first doses of ipilimumab and nivolumab resulted in LCV after 14 days. The presence of neutrophils in and around the lumens of the perivascular plexus and the lack of immunoglobulins (IgG, IgM, IgA), C3, and complement component 1q (C1q) depositions were shown using punch biopsies. Laboratory results only presented slightly positive ANCA. When cutaneous small vessel vasculitis clinically appeared, immunotherapy was discontinued and prednisone (1 mg/kg daily) was initiated. However, this patient died from disease progression after 9 weeks (19).

Another case was included within a systematic review of reports of immune-related CNS vasculitis following checkpoint inhibitors (20). A man with metastatic RCC, who received second-line nivolumab following progression on pazopanib, was admitted to the hospital due to dizziness, apraxia, and cognitive deficits on day 14 after the first dose of immunotherapy. This patient was diagnosed with secondary vasculitis using brain MRI, which showed microvascular hemorrhage. Initially, he was treated with methyl prednisolone, which was replaced with prednisolone when his condition improved. Afterward, the patient was readmitted with worsening apraxia and confusion resulting from vasculitis relapse. He died six months after nivolumab administration.

Although vasculitis is a relatively rare complication of immune checkpoint inhibitors, its early diagnosis and treatment are crucial for improving the patient*’*s prognosis. Managing the condition requires close cooperation between an oncologist and neurologist. The first step should be withholding immunotherapy. As part of the differential diagnosis, it is necessary to exclude cancer progression within the CNS, infectious causes of the present symptoms and/or signs, ischemic stroke, and radio-necrosis. Erritzøe-Jervild and collaborators recommended contrast-enhanced brain MRI, angiography, and CSF testing analysis for routine diagnostic workups of suspected cerebral vasculitis (20). Treatment is based on corticosteroids (methyl prednisolone i.v. up to 1 g daily for 3–5 days with or without IVIG), but rituximab or plasmapheresis may also be considered in refractory cases (21). For treatment response assessments, monitoring the absolute eosinophil counts, serum CRP, and MRI findings could be useful. Some authors consider monitoring CSF pleocytosis to also be critical (20).

When assessed in relation to the proposed diagnostic framework for neurological immune-related adverse events (nirAEs), the present case fulfills criteria for possible CNS vasculitis. Although CSF analysis and angiographic or histopathologic confirmation were not available, the temporal association with immune checkpoint inhibition, compatible neuroimaging, exclusion of common stroke mechanisms, and clinical as well as paraclinical response to corticosteroids support an immune-mediated process. It should be acknowledged that current nirAE criteria are not yet validated and may be relatively stringent; in recent case series, including that of Erritzøe-Jervild et al., most cases similarly fulfilled only *‘*possible*’* criteria. Future refinements of diagnostic frameworks may consider incorporating structured treatment response and inflammatory marker dynamics as supportive elements.

## 4 Conclusions

In cancer patients treated with checkpoint inhibitors, clinically significant possible supratentorial vasculitis is a rare immune-related adverse event that might lead to discontinuing systemic therapy. To improve patient outcomes, early diagnosis and timely immunosuppressive treatment are essential.

## Data Availability

The original contributions presented in the study are included in the article/supplementary material. Further inquiries can be directed to the corresponding author.
